# Influence of secretome from porcine cardiosphere-derived cells on porcine macrophage polarization and their possible implications for cardiac remodeling post-myocardial infarction *in vitro*

**DOI:** 10.3389/fcell.2025.1601743

**Published:** 2025-06-30

**Authors:** M. Pulido, M. A. de Pedro, A. M. Marchena, V. Alvarez, J. G. Casado, F. M. Sanchez-Margallo, E. López

**Affiliations:** ^1^ Stem Cell Therapy Unit, Jesús Usón Minimally Invasive Surgery Centre, Cáceres, Spain; ^2^ RICORS-TERAV Network, ISCIII, Madrid, Spain; ^3^ Immunology Unit, University of Extremadura, Cáceres, Spain; ^4^ Institute of Molecular Pathology Biomarkers, University of Extremadura, Cáceres, Spain

**Keywords:** cardiosphere-derived cells (CDCs), secretome, macrophage polarization, regenerative medicine, pro-reparative macropahge

## Abstract

The inflammatory response plays a crucial role in tissue repair following myocardial infarction (MI), with macrophages being central regulators of inflammation and tissue remodeling. Macrophage polarization between pro-inflammatory M1 and anti inflammatory M2 phenotypes significantly influences inflammation and tissue repair. This study evaluates the effect of the secretome from porcine cardio sphere-derived cells (S-CDCs) on macrophage polarization and its downstream impact on endothelial cells (HUVECs) and cardiac fibroblasts (PCF). Macrophages were treated with the secretome from S-CDCs, and their polarization status was assessed. Conditioned media from treated macrophages were applied to HUVECs and PCFs to evaluate effects on migration, wound healing, and fibrotic activity. Additionally, transcriptomic profiling of S-CDCs was performed to identify relevant cytokines. S-CDCs induced a mixed M1/M2 phenotype in macrophages, attenuating M1-associated inflammation without fully promoting M2 characteristics. Conditioned medium from S-CDC-treated M1 macrophages enhanced migration and wound healing in HUVECs, indicating proangiogenic effects. In contrast, medium from M2 macrophages did not show similar activity. Additionally, S-CDC-treated M1 macrophage medium modulated the migratory and fibrotic behavior of PCFs. Transcriptomic analysis revealed a cytokine profile enriched in pro-reparative factors such as VEGFA, TGFB, and CCL2. These findings suggest that S-CDCs modulate macrophage polarization to promote tissue repair and angiogenesis while minimizing excessive inflammation. This highlights their potential as a therapeutic strategy to enhance cardiac regeneration following MI.

## 1 Introduction

The inflammatory response plays a crucial role in the outcome of myocardial infarction (MI). Cell death from myocardial ischemia recruits and activates immune cells, triggering an early inflammatory phase followed by tissue repair and scar formation (Prabhu and Frangogiannis, 2016; Kubota and Frangogiannis, 2022). In this process, macrophages are an essential component of innate immunity, playing a pivotal role (Mouton et al., 2018; Duncan et al., 2020), exhibiting substantial heterogeneity and plasticity with M1 and M2 phenotypes (Martinez et al., 2008).

The inflammatory response, promoted by the pro-inflammatory M1 phenotype, facilitates the removal of necrotic cells and tissue remodeling. However, an excessive inflammatory response can exacerbate tissue damage by secreting cytokines and pro-inflammatory factors that induce apoptosis and cellular dysfunction. Multiple studies have demonstrated that elevated pro-inflammatory cytokine production in the heart correlates with worsening outcomes (Frangogiannis, 2014; Fang et al., 2015; Westman et al., 2016; Zhang and Dhalla, 2024). In mice, TNFα levels increased significantly 1 day after MI, and deleting TNFα led to a marked improvement in myocardial function 3 days after the MI (Zhang et al., 2013). Similarly, inhibiting IL-1α in mice with left anterior descending artery ligation and reperfusion decreased inflammasome formation, reduced infarct size, and helped maintain left ventricular function (Mauro et al., 2017).

In contrast, M2 macrophages, with an anti-inflammatory phenotype, promote tissue repair and angiogenesis through the secretion of growth factors and anti-inflammatory cytokines. This M2 polarization facilitates the activation of fibroblasts as well as the formation of new blood vessels through interactions with endothelial cells, which are vital for cardiac tissue repair and remodeling after MI (Jian et al., 2023). However, while initial reparative fibrosis is vital for preventing ventricular wall rupture, excessive fibrosis in and around the infarcted areas can cause an oversized scar. This excessive scarring progressively impairs heart function and could ultimately lead to heart failure. Therefore, managing fibrosis is key to improving MI outcomes (van den Borne et al., 2010; Shinde and Frangogiannis, 2014).

Increasing evidence indicates that macrophages are involved in regulating tissue damage through their secretome, modulating inflammation in their microenvironment. Additionally, damaged tissue cells can release factors that activate macrophages, creating an interaction loop that regulates both, macrophage switching and tissue cell response to injury (Wang et al., 2017; Liu et al., 2020; Jian et al., 2023). Thus, targeting macrophage therapy is becoming a real therapeutic strategy in the field of cardiovascular disease (Xu et al., 2022).

In the field of cardiovascular disorders, cardiosphere-derived cells (CDCs), which consist of mesenchymal, stromal, and progenitor cells derived from myocardial biopsy cultures (Messina et al., 2004; Davis et al., 2009), along with their secretome, are emerging as promising therapeutic options. While CDCs induce cardiac reparative mechanisms, their minimal direct cardiomyogenic differentiation does not significantly contribute to their beneficial effects (Tang et al., 2010). Instead, their therapeutic effects are mainly due to paracrine mechanisms mediated by their secretome, which consists of a complex array of soluble molecules that include cytokines, chemokines, cell adhesion molecules, lipid mediators, growth factors, and extracellular vesicles (EVs) (Xue et al., 2024). These paracrine actions have an impact on cardiac macrophages, influencing their behavior and contributing to tissue repair (Chimenti et al., 2010; de Couto et al., 2017).

Consistent with this, previous studies from our group have demonstrated *in vivo* the regulatory role of EVs from CDCs (EV-CDCs) on macrophage and neutrophil polarization. In porcine models of MI, the administration of EV-CDCs promotes macrophage polarization towards the M2 phenotype, counteracting the excessive inflammatory response usually observed in the acute phase of MI (Blázquez et al., 2016; López et al., 2020). However, the specific biological mechanisms involved and the overall effect of the secretome released by CDCs are still unknown.

Therefore, in the present study, we sought to unravel the impact of secretome from porcine CDCs (S-CDCs) on porcine macrophage polarization and its potential implications in endothelial cells and fibroblasts, which are crucial for tissue repair. We observed that S-CDCs may influence macrophage polarization, promoting a shift toward a more reparative profile compared to untreated M1 macrophages. In addition, our findings suggest that modulating macrophage polarization could influence their interactions with other cells involved in post-MI remodeling, highlighting the potential of S-CDCs as a supportive strategy to enhance cardiac repair post-MI.

## 2 Materials and methods

This study has been reported in accordance with the ARRIVE Guidelines for reporting experiments involving animals. All protocol was approved by the Jesús Usón Minimally Invasive Surgery Centre Animal Care and Use Committee (Ref 002/21) and the Extremadura Regional Government (EXP-20220329), and it complied fully with the Directive 2010/63/EU of the European Parliament on the protection of animals used for scientific purposes.

**FIGURE 1:**
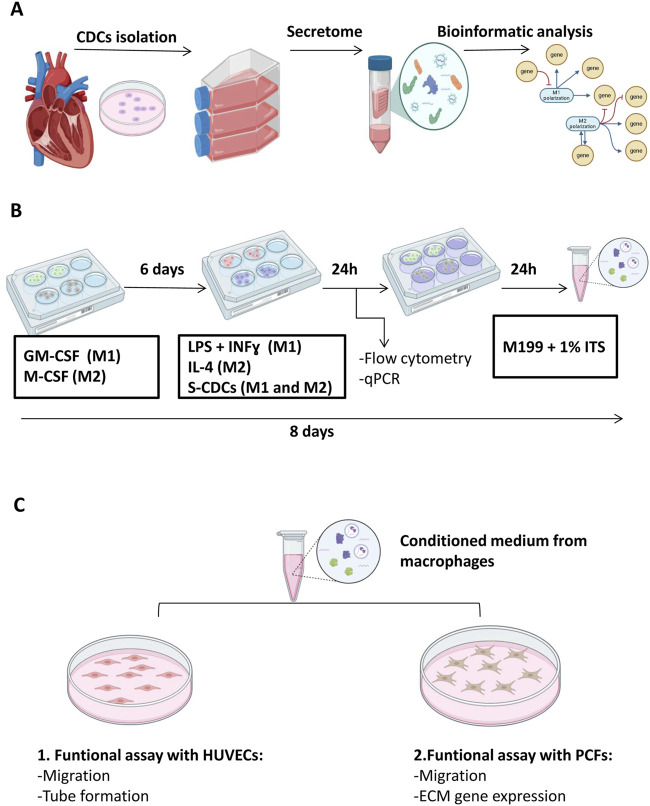
Workflow of materials and methods. **(A)** Isolation and bioinformatic analysis of secretome from CDCs. **(B)** Differentiation and treatment of macrophages with S-CDCs **(C)** Functional assays with conditioned medium from macrophages: **(C1)** Interaction with endothelial cells and **(C2)** interaction with cardiac fibroblasts. CDCs: cardiosphere-derived cells; S-CDCs: secretome released by cardiosphere-derived cells. HUVECs: Human umbilical vein endothelial cells. PCF: porcine cardiac fibroblasts. This figure was created with BioRender.com.

The workflow of the protocol performed in this study is summarized in Figure 1.

### 2.1 Isolation and characterization of secretome from CDCs

CDCs (n = 4) were isolated from auricular explants of four separate Large White pigs as previously described (Álvarez et al., 2022). Briefly, cardiac tissue explants were mechanically disaggregated and digested three times with a solution of 0.2% trypsin (Lonza, Basel, Switzerland), and 0.2% collagenase IV (Sigma-Aldrich, St. Louis, MO, United States).

Prior to secretome extraction, the identity of CDCs was confirmed by flow cytometry analysis assessing the expression of standard surface markers, including positive expression for CD117, Sca-1, and CD90, and low expression of hematopoietic marker CD45. In addition, CDCs demonstrated multipotent differentiation potential towards adipogenic, chondrogenic, and osteogenic lineages, as shown by specific staining methods (Data not shown).

CDCs at a confluence of 80% at passages 12–15 were used to obtain the S-CDCs. Culture medium was replaced by secretome isolation medium (1% insulin-transferrin-selenium (Thermo Fisher Scientific Inc., Waltham, MA, United States) in DMEM (Sigma Aldrich, MO United States) with 1% Penicillin/Streptomycin (Gibco, MA, United States). The supernatants were collected at day four and centrifuged in two steps: first at 1,000 × *g* for 10 min, and then 5,000 × *g* for 20 min at 4°C. Supernatants were filtered through a 0.22 μM filter to eliminate dead cells and debris, and ultra-filtered through a 3 kDa MWCO Amicon® Ultra device (Merck-Millipore, MA, United States) at 4,000 × *g* for 40 min at 4 °C. The concentration of proteins from enriched secretomes was quantified by a Bradford assay (Bio-Rad Laboratories, Hercules, CA, United States). Finally, the S-CDCs were stored at −80°C for further analysis.

For EV-CDCs isolation, secretome samples from the four CDC lines were equally pooled according to protein concentration (n = 4). The concentrated secretome pool was ultracentrifuged at 110,000 × *g* for 2 h in a MAX-XP ultracentrifuge equipped with a TLA-45 fixed-angle rotor (Beckman Coulter, Krefeld, Germany), and the resulting pellet was washed with 0.1 µm filtered PBS (1 mL) and centrifuged again with the same settings. Finally, the supernatant was removed, and the isolated EV-CDCs were suspended in 50 µL of filtered PBS and stored at −80°C for further analysis.

The EV-CDCs present in the secretome were characterized by several techniques following the recommendations of the International Society for Extracellular Vesicles (ISEV) (Welsh et al., 2024). EVs morphological characteristics were confirmed by transmission electron microscopy (TEM) using a Zeiss EM 900 at 80 kV, equipped with a 2k slow scanning CCD camera (TRS), while the particle size and concentration were estimated by nanoflow cytometry (nFC). A NanoAnalyzer equipped with a 488 nm laser and two single photon counting avalanche photodiodes (ADP) (NanoFCM, Inc., Nottingham, United Kingdom) with calibration settings used in the EV Core Facility Marburg and its previously described protocol (de Pedro et al., 2023) was used for this estimation. All samples were analyzed using NF Profession V2.0 software (NanoFCM, Inc.).

### 2.2 Isolation, differentiation and co-culture of monocytes-derived macrophages and secretome from CDCs

Approximately 12 mL of venous blood was collected from Large White pigs into EDTA-3K tubes (BD Biosciences, San Jose, CA, United States). Peripheral blood mononuclear cells (PBMCs) were isolated using Ficoll-Paque Plus (GE Healthcare, Chicago, IL, United States) density gradient centrifugation for 20 min at 1,200 *g*. Six-well plates were pre-treated with 2 mL of porcine serum and incubated at 37°C for 1 h.

PBMCs were then cultured in RPMI-1640 medium (Sigma Aldrich) supplemented with 10% Fetal Bovine Serum (FBS, Gibco), 1% Penicillin/Streptomycin, and 1 mM glutamine. The cells were plated on the pre-treated plates and incubated for 24 h at 37°C with 5% CO2. After this period, non-adherent cells were removed by washing four times with fresh RPMI-1640 medium.

For monocyte-to-macrophage differentiation, the adherent cells were cultured in complete RPMI-1640 medium with 5% porcine serum supplemented with 50 ng/mL recombinant human GM-CSF (Miltenyi Biotec, Bergisch Gladbach, Germany) for M1 macrophage differentiation or 50 ng/mL recombinant human M-CSF (Miltenyi Biotec) for M2 macrophage differentiation. This process was conducted over 7 days.

For macrophage activation, on day 6 of culture, 100 ng/mL LPS (Sigma Aldrich) and 100 ng/mL IFN-gamma (Raybiotech, GA, United States) were added to induce M1 activation, while 100 ng/mL IL-4 (Raybiotech) was used for M2 activation. This protocol has been previously published (Pulido et al., 2022). Simultaneously, activated macrophages were treated with 100 μg/mL of S-CDCs, while untreated M1 and M2 macrophages were treated with the secretome isolation medium.

On day 7, macrophages were detached by incubating with 1 mL PBS-EDTA (5 mM, pH 7) for 10 min at 37°C with 5% CO2 for flow cytometry and qPCR analysis. For the conditioned medium collection, a parallel experiment was performed in which, on day 7, the medium was replaced with secretome isolation medium for 24 h to eliminate the effect of the secretome and focus solely on what was released by the macrophages. After these 24 h, the conditioned medium was collected. The protein concentration of the culture supernatants was measured using the Bradford assay, following the manufacturer’s instructions.

### 2.3 Cell culture for Human Umbilical Vein Endothelial Cells (HUVECs) and porcine cardiac fibroblats (PCFs)

HUVECs (ATCC) were cultured in Endothelial Cell Basal Medium (EBM-2, Lonza) supplemented with growth factors (EGM-2 SingleQuotsTM, Lonza) and used at a maximum passage number of five.

For the isolation and culture of PCFs, hearts from experimental Large White pigs were perfused with ice-cold PBS through the left ventricle, and the cardiac tissue was finely sectioned and plated in 25 cm^2^ cell culture flasks with DMEM/F12 (VWR) supplemented with 10% FBS, 1% glutamine, and 1% Penicillin/Streptomycin. Cells were allowed to migrate from the tissue, with tissue remnants removed on the second day. The adherent cells were cultured until they reached 70%–80% confluency and a spindle-shaped morphology, typically around 10 days post-isolation. Fibroblast identity was confirmed by immunostaining for vimentin and fibroblast-specific marker (clone D7-FIB) (Data not shown).

### 2.4 Flow cytometry

Direct immunofluorescence study was performed with the antibodies, CD163 (clone 2A10/11, BD Pharmingen, CA, United States), CD206 (clone MMR, Invitrogen, MA, United States), and SLA-II (clone 2E9/13, Bio-Rad).

A total of 2 × 10^5^ cells were incubated for 30 min at 4°C with adequate concentrations of monoclonal antibodies and then washed and re-suspended in PBS. The analysis was performed in a FACScalibur cytometer (BD Biosciences) after the acquisition of 10^5^ events. First, cells were selected using forward and side scatter parameters, and then, were characterized by their fluorescence using CellQuest software (BD Biosciences). In all experiments, appropriate isotype-matched negative controls were included.

### 2.5 qPCR

Total RNA from macrophages and PCFs were purified using PureLink™ RNA Mini Kit (Thermo-Fisher Scientific Inc.), following the manufacturer’s protocol for total RNA extraction. The quality and concentration of total RNAs were evaluated by Implen NanoPhotometer® (Thermo Fisher). For each RNA sample, 300 ng of the corresponding cDNA was synthesized using iScript Reverse Transcription Supermix (BioRad), according to the manufacturer’s instructions. A volume of 2 μL of cDNA for each sample was then employed as a template for the qPCR amplification with the TaqMan™ Fast Advanced Master Mix (Cat. 4444964, Thermo-Fisher Scientific Inc.). Commercial TaqMan® Gene Expression Assays probes (Thermo-Fisher Scientific Inc.) were used, according to the manufacturer’s recommendations, to evaluate the relative expression of the following genes: *TNFA* (Ss03391318_g1*)*, *IL10* (Ss03382372_u1), *VEGFA* (Ss03393993_m1), *ACTA2* (Ss04245588_m1), *COL1A1* (Ss03373340_m1) and *COL3A1* (Ss04323794_m1). Samples were evaluated in duplicate and 2 μL of water was substituted by templates to perform negative control for each probe. The qPCR reaction was performed in a QuantStudio 3 Real-Time PCR System (Applied Biosystems, Thermo Fisher Scientific Inc.), and the products were quantified by fluorescent method using 2^−ΔΔCT^ expression with *HPRT1* (Ss03388274_m1) as endogenous control. All data were analyzed in the Thermo Fisher Cloud (also called Thermo Fisher Connect).

### 2.6 Wound healing assay

An artificial wound was created in HUVEC and PCFs on an 80%–90% confluent cell monolayer in a 24-well plate using a 200 μL pipette tip. Mitomycin C (Sigma-Aldrich) at 5 μg/mL was added to the culture wells to eliminate the influence of cell proliferation. The effects of 100 μg/mL conditioned medium from basal or secretome-treated macrophages on cell migration were monitored by microscopy at 0 and 24 h. Images were acquired using an inverted microscope (Nikon Elipse TE2000-S) and analyzed by ImageJ. The number of migrated cells was counted in the wounding zone determined by a predefined frame.

### 2.7 Migration assay

HUVEC and PCFs migration were examined using a Boyden chamber of 6.5-mm polycarbonate membrane with 5 μm pores (Costar, Corning, NY, United States). A total of 5 × 10^4^ cells were added to the upper compartment and the bottom chambers of Transwell were filled with 100 μg/mL concentration of conditioned medium from basal or secretomes-treated macrophages. After 24 h of incubation, cells on the upper side of the membrane (non-migrated cells) were scraped with a cotton ball, and cells spreading on the bottom side of the membrane (invasive cells) were fixed with methanol (Cromakit, Granada, Spain) and stained with eosin-thiazine (Cromakit, Granada, Spain). Images were taken by an inverted microscope (Nikon Elipse TE2000-S) and analyzed using ImageJ with Cell Counter plug-in.

### 2.8 Tube formation assay

HUVECs tube formation capacity was analyzed by using an Angiogenesis μ-slide system (IBIDI GmbH, Planegg/Martinsried, Germany). μ-slide wells were coated with 10 μL Growth Factor Reduced (GFR) Matrigel (BD Biosciences) for at least 30 min at 37°C. After Matrigel polymerization, endothelial cells at a density of 2 × 10^4^ were plated and incubated at 37°C for 24 h in the presence of 100 μg/mL of conditioned medium from basal or secretomes-treated macrophages. Images were taken with an inverted microscope (Nikon Elipse TE2000-S) and analyzed by using ImageJ Software with Angiogenesis Analyzer plug-in.

### 2.9 Transcriptomic analyses

Transcriptomic analyses were performed to evaluate the potential cytokines through which the secretome modulates macrophages response.

The whole transcriptomic sequencing process was performed by Arraystart (Arraystart, Rockville, MD, United States). Briefly, total RNA from the four S-CDC samples was isolated with mirVana™ miRNA Isolation Kit (Thermo Fisher Scientific Inc.), quantified using Nanodrop, and qualified by agarose gel electrophoresis. The mRNA is enriched using oligo (dT) magnetic beads. RNA-seq libraries were prepared using the KAPA Stranded RNA-Seq Library Prep Kit (Illumina, San Diego, CA, United States), qualified with the Agilent 2,100 Bioanalyzer, and quantified by the qPCR absolute quantification method. Sequencing was performed with Illumina NovaSeq 6,000.

Image analysis and base calling were performed using Solexa pipeline v1.8 (Off-Line Base Caller software, v1.8). Sequence quality was examined using the FastQC software. The trimmed reads (trimmed 5′, 3′-adaptor bases using cutadapt (Martin, 2011)) were aligned to reference genome (*sus scrofa*) using Hisat2 software (Kim et al., 2015). The transcript abundances for each sample were estimated with StringTie (Pertea et al., 2015), and the FPKM (Mortazavi et al., 2008) value for gene level was calculated with R package Ballgown (Frazee et al., 2015).

We selected 16 cytokines based on their established involvement in macrophage polarization and tissue remodeling, including TNFA, IFNG, TLR4, CSF2, IL10, IL4, IL13, TGFB1, TGFB2, CCL24, CSF1, IL1B, VEGFA, CXCL14, IL33, and CCL2. To assess their potential expression, we analyzed transcriptomic data obtained from S-CDCs (S-CDCs. A cytokine gene was considered expressed if its mRNA was detected in at least three out of four samples. While mRNA detection does not directly confirm protein secretion, this approach allowed us to identify key pro-reparative factors likely produced by S-CDCs An interaction network depicting the roles of these cytokines in M1 and M2 macrophage polarization was generated using Cytoscape 3.8.2.

### 2.10 Statistical analysis

Data were statistically analyzed with GraphPad Prism (version 8.0). For analysis of differences between the two groups, Student’s t-test was performed. Data are presented as mean ± SD considering at least three independent replicates for each assay. The p values ≤0.05 were considered statistically significant. In all cases: *p < 0.05, **p < 0.005.

## 3 Results

### 3.1 Effect of S-CDCs treatment on the inflammatory response of porcine macrophages

Macrophages play a critical role in both the progression and resolution of MI. Their dynamic polarization into pro-inflammatory (M1) and anti-inflammatory or reparative (M2) phenotypes significantly influence local inflammation, ECM remodeling, and tissue repair. To investigate how the secretome, enriched in EVs (Supplementary Figure S1), might influence macrophage polarization and inflammatory responses, we analyzed the effects of secretome treatment on activated macrophages.

Surface marker analysis by flow cytometry was performed on macrophages treated with 100 μg/mL of secretome for 24 h. We evaluated the percentage of positive macrophages and the mean fluorescence intensity (MFI) for SLA-II, CD206, and CD163 to assess changes in their polarization state.

**FIGURE 2:**
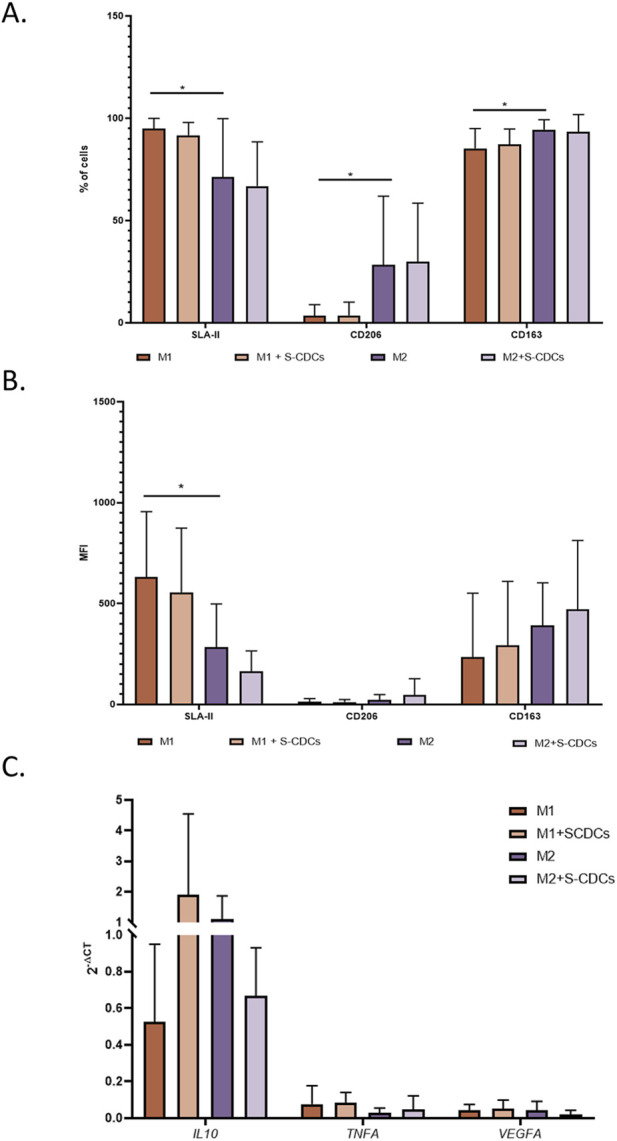
Analysis of macrophage markers and cytokine expression following S-CDC treatment. Throughout the figure: untreated M1 macrophages (orange), M1 treated with S-CDCs (light orange), M2 macrophages (purple) and untreated M2 treated with S-CDCs (light purple). **(A)** Percentage of cells positive for SLA-II, CD206, and CD163 markers as measured by flow cytometry (n = 9). **(B)** Mean fluorescence intensity (MFI) of SLA-II, CD206, CD163 measured by flow cytometry (n = 9). **(C)** Quantitative PCR analysis of *IL10*, *TNFA*, and *VEGFA* gene expression levels (n = 4). Statistical analysis was conducted using Student’s t-test. Data are presented as mean values, and error bars indicate SEM. Statistical significance is denoted by asterisks: *p < 0.05 **p < 0.005.

Although no statistically significant differences were found in the percentage of positive cells or MFI for any of the markers analyzed, we observed a slight decrease in SLA-II expression and a slight increase in CD163 in both the percentage of positive cells (Figure 2A) and MFI (Figure 2B) for M1 macrophages treated with S-CDCs compared to untreated M1 macrophages. Similarly, for M2 macrophages, there was also a slight decrease in SLA-II expression (both in percentage and MFI) and an increase in MFI of CD163 marker (Figures 2A,B).

To further investigate the effects of the secretome on macrophage gene expression in the context of MI, we analyzed *IL10*, *VEGFA*, and *TNFA* expression levels by qPCR. These cytokines are associated with tissue repair, angiogenesis, and tissue damage exacerbation, respectively. The qPCR results did not reveal any statistically significant differences between treated and untreated macrophages (Figure 2C). Nonetheless, we observed non-significant trends in gene expression that may suggest changes in macrophage activation states. In M1 macrophages, there was a tendency toward increased expression of IL10, VEGFA, and TNFA, which could be indicative of a mixed activation profile with features of both tissue repair and inflammatory signaling. Conversely, in M2 macrophages, we noted a non-significant decrease in IL10 and VEGFA, along with a slight increase in TNFA, which may reflect a shift toward a more pro-inflammatory and less reparative phenotype.

### 3.2 Effect of S-CDCs treatment on the pro-angiogenesis capacity of porcine macrophages: In vitro assays with HUVECs

**FIGURE 3:**
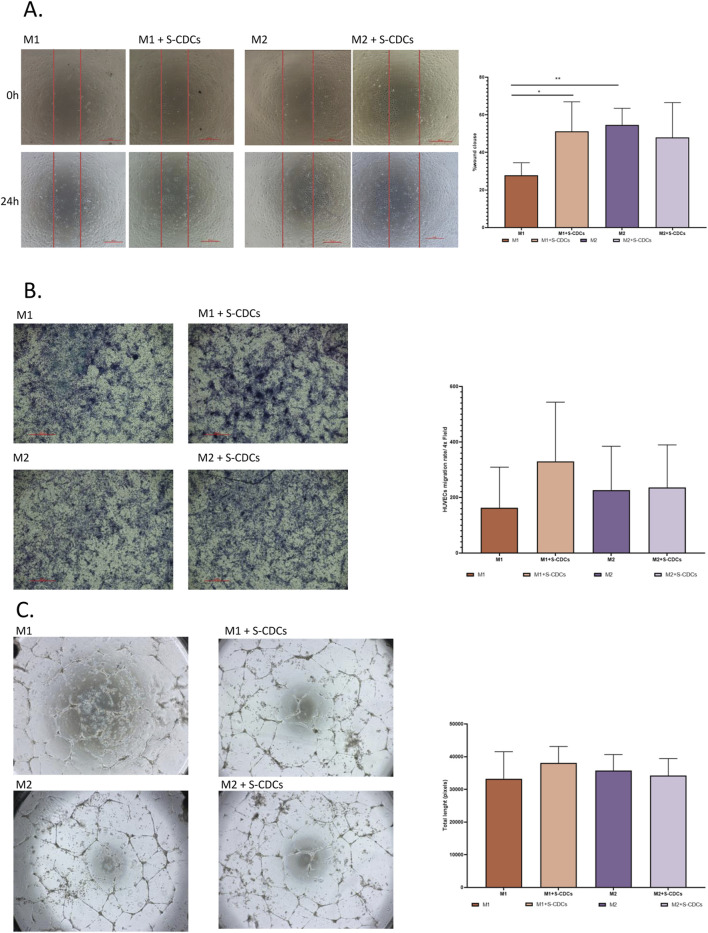
Effect of S-CDCs treated macrophages on HUVECs. For the whole figure: untreated M1 macrophages (orange), M1 treated with S-CDCs (light orange), untreated M2 macrophages (purple) and M2 treated with S-CDCs (light purple). **(A)** Percentage of wound closure was measured by wound healing assay (n = 4). **(B)** Migration assay results (n = 4). **(C)** Tube formation assay measured by tube length pixels (n = 5). Statistical analysis was conducted using Student’s t-test. Data are presented as mean values, and error bars indicate SEM. Statistical significance is denoted by asterisks: *p < 0.05 **p < 0.005.

Since angiogenesis is crucial for the healing response following a MI, we aimed to investigate how S-CDCs could influence this process by modulating macrophage function, evaluating the angiogenic capacity of HUVECs through wound healing, migration, and tube formation assays. The results showed that HUVECs treated with conditioned medium from S-CDC-treated M1 macrophages exhibited increased cell migration with a significant effect in the wound healing assay (Figure 3A) and enhanced migration in the cell migration assay (Figure 3B), reaching similar levels to those observed with M2 macrophages conditioned medium treatment, indicating a notable pro-angiogenic effect. However, S-CDCs had no significant impact on M2 macrophage-conditioned medium. Although a trend towards enhanced tube formation was observed in HUVECs treated with conditioned medium from S-CDC-treated M1 macrophages (Figure 3C), this was not statistically significant compared to those treated with medium from untreated M1 macrophages. Similarly, no enhancement of pro-angiogenic effects was observed in HUVECs treated with conditioned medium from treated M2 macrophages.

### 3.3 Effect of porcine macrophages treated with S-CDCs on the migration and fibrotic capacity of cardiac fibroblast

Additionally, we analyzed the impact of treatment with S-CDCs on macrophages and their interaction with cardiac fibroblasts, which play a crucial role in MI remodeling through extracellular matrix deposition and scar formation. To assess this effect, preconditioned mediums of activated macrophages were used to treat PCFs.

**FIGURE 4:**
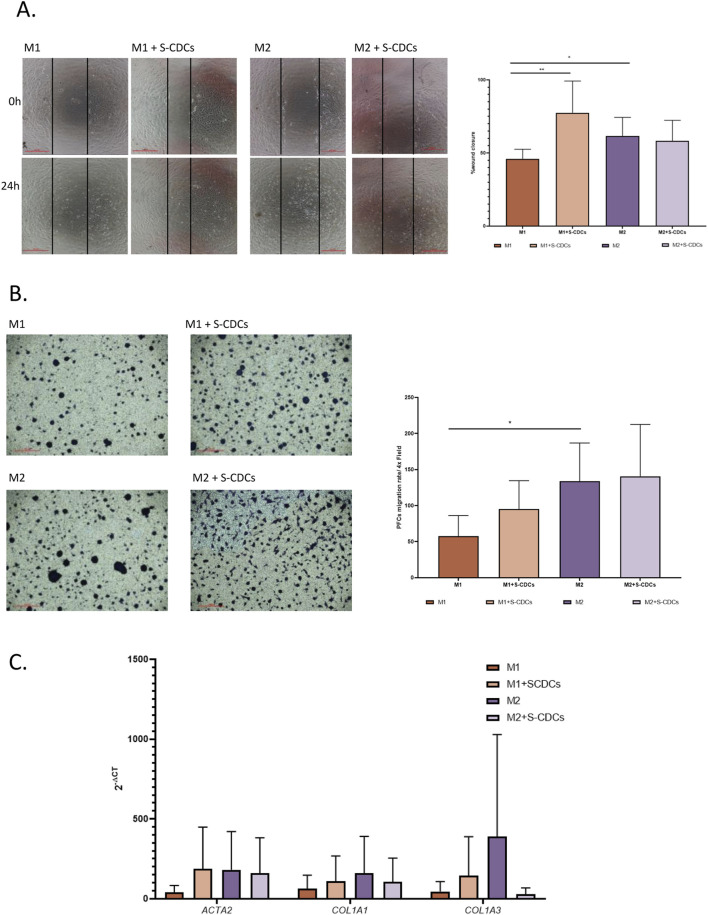
Effect of S-CDC-treated macrophages on PCFs. For the figure panels: untreated M1 macrophages (orange), M1 treated with S-CDCs (light orange), untreated M2 macrophages (purple) and M2 treated with S-CDCs (light purple). **(A)** Percentage of wound closure was measured by wound healing assay (n = 5). **(B)** Migration assay results (n = 4). **(C)** Gene expression levels of *ACTA2*, *COL1A1* and *COL3A1* measured by qPCR (n = 3). Statistical analysis was conducted using Student’s t-test. Data are presented as mean values, and error bars indicate SEM. Statistical significance is denoted by asterisks: *p < 0.05 **p < 0.005.

The activation capacity of these PCFs was evaluated by measuring wound healing and migration. The results showed that PCFs treated with conditioned medium from S-CDC-treated M1 macrophages exhibited significantly enhanced wound healing (Figure 4A) and increased cell migration (Figure 4B) compared to PCFs treated with conditioned medium from untreated M1 macrophages, with the increase even surpassing the effect observed with M2 macrophages. This indicates a clear stimulatory effect of S-CDCs. In contrast, conditioned medium from S-CDC-treated M2 macrophages did not induce significant changes in wound healing or migration in PCFs when compared to medium from untreated M2 macrophages.

Subsequently, we aimed to assess how the secretome treatment affected the expression of profibrotic genes. To address this, we analyzed the expression of fibrotic genes *ACTA2*, *COL1A1*, and *COL3A1* by qPCR. Consistent with our previous findings, we observed a no significant increase in expression in PCFs treated with conditioned medium from S-CDCs-treated M1. However, the expression did not reach the levels observed in PCFs treated with conditioned medium from M2 (Figure 4C).

On the other hand, in PCFs treated with conditioned medium from M2 macrophages, although the results were not statistically significant, we observed a reduction in the expression of these genes following S-CDCs treatment. This aligns with our above observations, where conditioned medium from treated M2 macrophages not only failed to significantly activate PCFs compared to untreated-M2 macrophages but may also reduce their activity and matrix production (Figure 4C).

### 3.4 *In silico* analysis of S-CDCs cytokine profile: Implication of macrophages polarization

Given the observed effect of the secretome on enhancing pro-angiogenic and tissue-remodeling properties, we set out to investigate the potential mechanisms through which the secretome modulates these cellular responses. To this end, we conducted a transcriptomic analysis to identify genes encoding key cytokines involved in the regulation of inflammation, angiogenesis, and tissue repair.

We focused on pro-inflammatory factors, such as *TNFA*, *IFNG*, *TLR4*, and *CSF2* (Kan et al., 2016; Orecchioni et al., 2019; Kim et al., 2021), which promote M1 polarization and drive inflammatory responses. In contrast, we examined anti-inflammatory cytokines, including *IL10*, *IL4*, *Il13*, *TGFB1*, *TGFB2*, *CCL24*, *CSF1*, *IL1B*, *VEGFA*, *CXCL14*, and *IL33*, which are critical for resolving inflammation, ECM remodeling, and tissue repair (Wheeler et al., 2018; Li et al., 2019; Lv et al., 2020; Kiseleva et al., 2023; Wang et al., 2023). Additionally, we analyzed dual-function cytokines, such as *CCL2*, which recruits monocytes during the early phases of myocardial infarction and supports their transition to reparative phenotypes during later stages (Chen and Frangogiannis, 2021; Shen et al., 2024).

**FIGURE 5:**
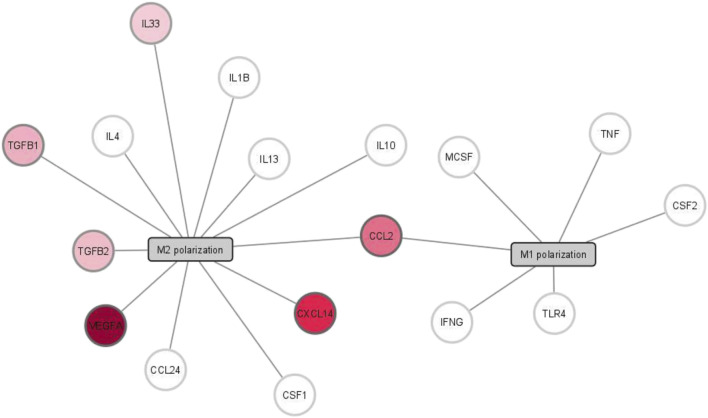
Interaction network between cytokines and macrophages. The network illustrates cytokines known to polarize macrophages towards M1 or M2 phenotypes. Genes encoding cytokines in the S-CDC are highlighted in shades of red, with color intensity representing relative abundance (pale for low abundance to deep red for high abundance). White nodes indicate cytokines not detected in the secretome.

The results of our analysis revealed a cytokine profile dominated by *VEGFA*, *TGFB1*, *TGFB2*, *CCL2*, *CXCL14*, and *IL33*, with *VEGFA* showing the highest expression (Figure 5). This cytokine profile aligns with the observed pro-angiogenic and tissue-remodeling effects of the secretome, suggesting a bias toward promoting tissue repair and angiogenesis. Interestingly, the absence of classical pro-inflammatory cytokines, such as *TNFA*, *IFNG*, and *TLR4*, indicates that the secretome likely minimizes M1 polarization and inflammatory responses. Similarly, the lack of canonical M2 activation cytokines, such as *IL10*, *IL4*, or *IL13*, suggests that the secretome does not induce a traditional M2 phenotype.

## 4 Discussion

Macrophages are central targets in various therapeutic strategies due to their high plasticity and ability to dynamically respond to changes in their microenvironment. These features allow macrophages to adopt distinct phenotypes, modulating their functions in response to local biochemical signals. In the context of MI, macrophages play a pivotal role, transitioning from a pro-inflammatory phenotype essential for initial tissue clearance to a reparative phenotype critical for cardiac remodeling and tissue healing. Given this dual role, a targeted manipulation of macrophage polarization may hold therapeutic potential for enhancing post-MI cardiac repair (Yin et al., 2024).

One promising approach involves the use of secretome, a complex mixture of proteins, lipids, and nucleic acids released by cells, which can influence the microenvironment and potentially guide macrophage polarization. In particular, the secretome derived from porcine CDCs contains bioactive factors capable of modulating immune responses and promoting tissue repair. Understanding how the S-CDCs affects macrophage phenotype and function *in vitro* could provide insights into its potential role in post-MI cardiac remodeling and identify mechanisms to fine-tune macrophage activity to optimize healing processes.

Isolation of peripheral blood monocytes is a well-established and widely used strategy for developing *in vitro* macrophage assays. In our initial set of experiments, we examined the effects of the secretome on the activation of *in vitro*-differentiated macrophages. Treatment with S-CDCs did not significantly alter the expression of surface markers or cytokines in any macrophage subpopulation compared to their respective untreated M1 or M2. Given that the macrophage activations were effective, with LPS + IFN-γ promoting M1 polarization and IL-4 enhancing M2 expression, the lack of effect from the secretome might be due to several factors. One possibility is that the concentration or specific bioactive components of the secretome are insufficient to lead marked phenotypic changes, unlike the robust, direct effects of LPS + IFN-γ or IL-4. However, although the observed changes were not statistically significant, some expression trends may suggest a partial modulation effect. A decrease in *SLA-II* and an increase in *CD163* could indicate a shift from a pro-inflammatory M1 state toward a more reparative M2-like profile. Likewise, slight, non-significant increases in *TNFA*, *IL10*, and *VEGFA* expression may point to a mixed activation state, combining both inflammatory and repair-associated signals. These preliminary observations, while not conclusive, may reflect a nuanced response in which the secretome influences macrophage behavior toward promoting tissue repair and resolution of inflammation without fully suppressing their immune activity (Meng et al., 2015).

It is well known that macrophages contribute to endothelial cell migration expressing several endothelial factors, such as vascular endothelial growth factor (VEGF), which is critical in tissue repair following an infarction (Glinton et al., 2022). The capacity of activated macrophages in terms of wound healing, migration, and tube formation was evaluated in a series of functional *in vitro* assays. Interestingly, our results demonstrated that the migration capacity of HUVEC increased when treated with a preconditioning medium derived from M1 macrophages co-cultured with S-CDCs. The enhanced migration of HUVECs in this condition reached comparable levels to the effects seen with M2 macrophages, which are known for their pro-healing and anti-inflammatory properties. This is consistent with fact that human primary M1 polarized macrophages can be re-polarized by secreted factors from their own counterparts, M2 macrophages, which are known for their pro-healing and anti-inflammatory properties, and *vice versa*, *in vitro* (Ploeger et al., 2012). Moreover, it has been recently demonstrated that secretome of macrophages can be internalized in HUVECs showing M2 high capacity to promote angiogenesis *in vitro* and *in vivo* after myocardial infarction (Guo et al., 2024).

The interplay between macrophages and fibroblasts is essential for scar formation and maturation, with different roles observed in the remodeling of the extracellular matrix (ECM) in cardiac tissue following MI (Mallikarjun et al., 2024). M1 macrophages are known for their initial pro-inflammatory response. In contrast, M2 macrophages facilitate ECM remodeling by secreting anti-inflammatory and tissue repair factors, which subsequently promote fibroblast proliferation and contribute to the resolution phase of inflammation and scar maturation. *In vitro* studies have demonstrated that M2 macrophages stimulate fibroblast activation by producing profibrotic factors, which significantly increase fibroblast proliferation and ECM deposition (Kurachi et al., 2021 Tan et al., 2024). However, in our study, the treatment of M1 macrophages treated with S-CDCs not only increased the migration of PCFs, but also led to an enhancement in wound healing, achieving an effect comparable to that of M2-polarized macrophages. This finding strongly suggests that S-CDC treatment modulates the profile of M1 macrophages, enabling them to adopt some reparative characteristics typically associated with M2 macrophages, thereby promoting early fibroblast activation and wound repair.

Furthermore, the observed possible increase in profibrotic gene expression in M1 macrophages treated with S-CDCs underscores the complexity of the M1 phenotype’s role in cardiac repair. Early activation of profibrotic pathways by M1 macrophages could be crucial for stabilizing the myocardial architecture immediately post-MI, as these cells initiate scar formation by releasing signals that attract fibroblasts and encourage ECM deposition (Kang et al., 2023).

Additionally, the profibrotic effect of M2-polarized macrophages is not enhanced by S-CDC treatment, which is beneficial, as it suggests that S-CDCs do not further stimulate M2-driven fibrosis beyond its baseline reparative role. This finding indicates that S-CDC treatment does not overly amplify the pro-fibrotic effects of M2 macrophages, thus avoiding the risk of excessive matrix deposition or scar rigidity.

The transcriptomic analysis further supported these observations by revealing a cytokine profile enriched in reparative factors, such as *VEGFA*, *TGFB1*, *TGFB2*, *CXCL14*, and *IL33*. This profile suggests that the secretome promotes an environment conducive to tissue repair, with a particular focus on angiogenesis and ECM remodeling. The absence of key pro-inflammatory cytokines like *TNFA*, *IFNG*, and *TLR4*, which are associated with M1 polarization, suggests that the secretome may minimize M1 activation and its associated inflammatory responses. Additionally, the lack of canonical M2 cytokines such as *IL10*, *IL4*, or *IL13* further supports the idea that the secretome does not fully polarize macrophages toward a classical M2 phenotype. Instead, it may induce a functional shift in macrophages that encourages reparative processes without following the conventional M1/M2 polarization axis.

This study provides evidence that S-CDC treatment may modulate macrophage phenotypes in ways that could support post-MI cardiac repair. S-CDCs significantly promoted endothelial cell migration by influencing macrophage activity to achieve similar effects to those of M2 macrophages, known for their reparative properties. Additionally, S-CDCs influence M1 macrophages to adopt reparative characteristics, supporting fibroblast activation, ECM remodeling, and wound healing without excessively amplifying the pro-fibrotic activity of M2 macrophages. The cytokine profile revealed by the transcriptomic analysis, together with the observed functional effects, suggests that the S-CDC secretome may create a pro-repair microenvironment that contributes to tissue regeneration while limiting inflammation or fibrosis. In conclusion, while further validation (particularly *in vivo*) is required, these findings suggests that S-CDCs may modulate macrophage activity supporting reparative processes following MI, highlighting their potential approach in cardiac regenerative strategies.

## Data Availability

The datasets presented in this study can be found in online repositories. The names of the repository/repositories and accession number(s) can be found below: https://www.ncbi.nlm.nih.gov/bioproject/PRJNA1217600, PRJNA1217600.

## Supplementary material

SUPPLEMENTARY FIGURE S1 EV-CDC characterization. **(A)** Electron microscopy EV-CDC preparations. **(B)** Sizing profile of representative pooled samples. Histograms represent the concentration of particles detected by nFC. EV-CDCs: extracellular vesicles released by cardiosphere-derived cells.

## Abbreviations

CDCs, porcine cardiosphere-derived cells; EVs, extracellular vesicles; EV-CDCs, extracellular vesicles from cardiosphere-derived cells; HUVECs, Human Umbilical Vein Endothelial Cells; MI, myocardial infarction; PBMCs, Peripheral blood mononuclear cells; PCFs, porcine cardiac fibroblasts; S-CDCs, secretome from porcine cardiosphere-derived cells.
