# Supplementary figures and images for: Influence of secretome from porcine cardiosphere-derived cells on porcine macrophage polarization and their possible implications for cardiac remodeling post-myocardial infarction in vitro
Source: Front Cell Dev Biol. 2025 Jun 30;13:1601743. doi: 10.3389/fcell.2025.1601743 (PMC12256530; doi:10.3389/fcell.2025.1601743)

## Slide 1
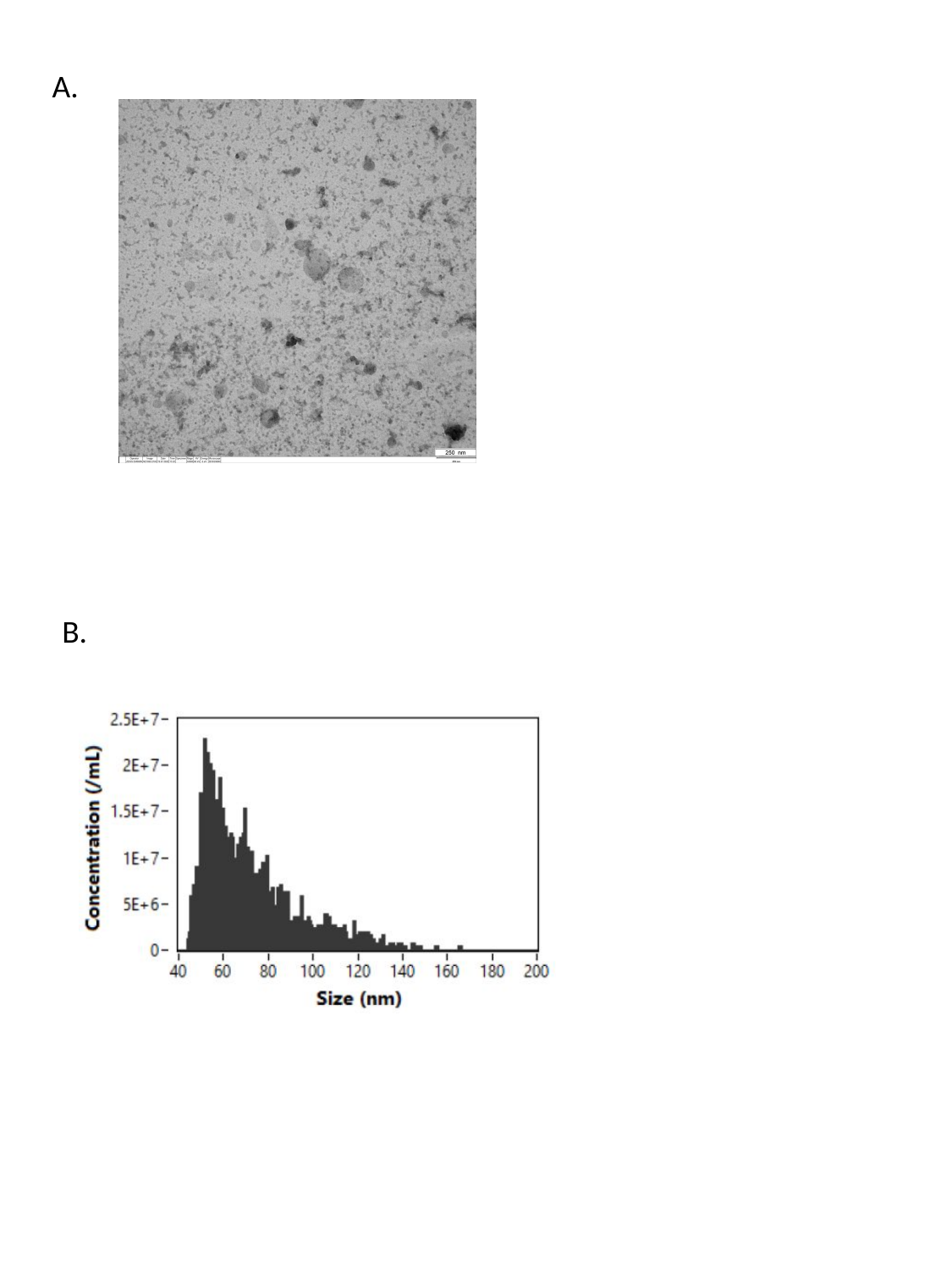

A.
B.

Supplement: Supplementary file 1 [file Presentation1.pptx]
